# Neural correlates of trait anxiety in sensory processing and distractor filtering

**DOI:** 10.1111/psyp.14706

**Published:** 2024-10-08

**Authors:** Michelle V. Faerman, Kaylena A. Ehgoetz Martens, Sean K. Meehan, W. Richard Staines

**Affiliations:** ^1^ Department of Kinesiology and Health Sciences University of Waterloo Waterloo Ontario Canada

**Keywords:** attention, distractor filtering, electroencephalography, event‐related potentials, inhibition, sensory gating, sensory processing, somatosensory, trait anxiety, visual

## Abstract

Evidence suggests that trait anxiety relates to cognitive processing and behavior. However, the relationships between trait anxiety and sensory processing, goal‐directed performance and sensorimotor function are unclear, particularly in a multimodal context. This study used electroencephalography to evaluate whether trait anxiety influenced visual and tactile event‐related potentials (ERPs), as well as behavioral distractor cost, in a bimodal sensorimotor task. Twenty‐nine healthy young adults completed the State–Trait Anxiety Inventory. Participants were directed to focus on either tactile or visual stimuli while disregarding the other modality, responding to target stimulus amplitude with a proportional grip. Previous research suggests that somatosensory N70 and visual P2 ERPs serve as markers of attentional relevance, with attention also impacting the visual P3 ERP. It was hypothesized that trait anxiety would modulate the ERPs susceptible to attentional modulation (tactile N70, visual P2 and P3) and not affect behavioral performance. Trait anxiety showed a large, significant interaction with attention for visual P3 latency in response to unimodal visual stimuli, with a positive relationship between P3 latencies and trait anxiety when attending toward the stimulus and negative when attending away. A large, positive main effect of trait anxiety on visual N1 amplitude for bimodal stimuli was also detected. As predicted, trait anxiety related to ERPs but not behavioral distractor cost. These findings suggest that trait anxiety modulates visual but not somatosensory processing correlates based on attention. The absence of overt behavioral performance effects suggests compensatory mechanisms may offset underlying differences in sensory processing.

## Abstract 2

For the first time in a crossmodal sensory context, event‐related potentials revealed a link between trait anxiety and task‐dependent sensory selection. Trait anxiety was related to reduced neural correlates of visual but not tactile distractor gating, and not sensorimotor performance. These findings shed light on modality‐specific sensory processing alterations resulting from individual differences in anxiety which did not manifest behaviorally.

## 1 INTRODUCTION

At a given moment, the central nervous system (CNS) processes an abundance of incoming (afferent) sensory information from the environment. To prevent higher cortical areas of the brain from information overload, a sensory gating mechanism regulates the access of task‐relevant and irrelevant information to higher levels of the CNS to optimize goal‐based action performance (Cromwell et al., 2008). The association between anxiety and a reduced ability to inhibit irrelevant distractors, otherwise known as inhibitory gating, has been well established in conditions with and without threat (Ansari & Derakshan, 2011a; Basten et al., 2011, 2012; Bishop, 2009; Stout et al., 2013). However, the effect individual differences in trait anxiety have on sensory gating in different stimulus modalities remains unclear. To date, evidence suggests that high trait anxiety has been linked to deficits in performance and efficiency of distractor inhibition (Basten et al., 2011; Bishop, 2009; Qi et al., 2013; Stout et al., 2013), response inhibition (Forster et al., 2015; Sehlmeyer et al., 2010; Xia et al., 2020), and other executive functions implicated in sensory gating and sensorimotor control (Ansari & Derakshan, 2011b; Hainaut & Bolmont, 2013), suggesting that there may be an association between trait anxiety and these fundamental processes that drive behavior.

Attentional control theory (ACT), developed by Eysenck et al. (2007), describes that anxiety modulates goal‐directed attentional control. High trait anxious individuals' attention is considered more heavily influenced by bottom‐up, stimulus‐driven neural processes than the top‐down, goal‐driven attentional system compared to low trait anxious individuals (Eysenck et al., 2007; Eysenck & Derakshan, 2011). This attentional bias observed in high trait anxious individuals is thought to be the consequence of an impaired central executive system (Eysenck et al., 2007; Eysenck & Derakshan, 2011) that acts on three lower level functions: inhibition, switching, and updating (Miyake et al., 2000). Another notable assumption drawn from ACT is that anxiety is associated with reduced processing efficiency more so than performance effectiveness (Eysenck et al., 2007). High trait anxious individuals tend to perform at a comparable level to those who are less anxious, presumably because of compensatory increases in neural effort (Eysenck et al., 2007). While these increases in neural effort signify a reduction of neural efficiency at performing the same task, particularly in cognitive tasks that require inhibition, switching, or updating, their overall performance ability is typically not hindered (see reviews by Berggren & Derakshan, 2012; Eysenck et al., 2007; Eysenck & Derakshan, 2011). Conversely, research has also shown that when the cognitive demands are sufficiently high, highly trait anxious individuals tend to show compromised performance compared to less anxious individuals (Basten et al., 2011; Bishop, 2009), such as a reduced ability to inhibit incongruent information in the color word Stroop task (Basten et al., 2011) and impaired inhibitory control in anti‐saccades (Ansari & Derakshan, 2011a). Sensory processing and sensorimotor integration draw upon the same executive functions impacted by trait anxiety. Therefore, it is highly plausible that trait anxiety may affect sensory processing and integration. However, few studies have addressed the relationship between trait anxiety and sensorimotor processes.

Sensory gating is considered a multistep process, with early stages occurring in the parietal and prefrontal neocortex (Yamaguchi & Knight, 1990) and later stages occurring in the hippocampus (Grunwald et al., 2003). Irrelevant sensory stimuli are “gated” out of one's attention earlier in the processing stream to prevent overloading higher cortical areas (Cromwell et al., 2008). In contrast, relevant stimuli are presumably processed to a greater extent. At early stages of sensory processing, one of the many functional roles of the prefrontal cortex is the gating of afferent sensory information (Yamaguchi & Knight, 1990). The dorsolateral prefrontal cortex (DLPFC) plays a crucial role in sensory gating, the goal‐directed control of attention, and inhibition of irrelevant information (Chao & Knight, 1995). The DLPFC was previously found to be hypoactivated in the left (Bishop, 2009) and hyperactivated in the right hemisphere (Basten et al., 2011, 2012; Forster et al., 2015) during cognitive tasks in healthy, highly anxious individuals. These anxiety‐dependent variations in prefrontal recruitment raise the possibility that sensory gating in highly anxious individuals may be compromised.

Sensory gating deficits have been previously found in clinically anxious populations, such as in individuals with post‐traumatic stress disorder (Holstein et al., 2010; Hunter et al., 2011), obsessive–compulsive disorder (Rossi et al., 2005), and panic disorder (Ludewig et al., 2002). Evidence suggests that sensory gating may also be impaired in subclinical high trait anxious populations (Chan et al., 2012; Duley et al., 2007). For example, prepulse inhibition (PPI) was impaired in high compared to low trait anxious individuals at rest (Duley et al., 2007). PPI, or startle reduction, refers to the attenuation of the startle reflex magnitude of a startling stimulus following exposure to a stimulus of low intensity (prepulse) (Duley et al., 2007). Respiratory sensory gating deficits (respiratory‐related evoked potential N1 peak gating ratios from second relative to first inspiratory occlusions) have also been found to be predicted by self‐reported measures of anxiety (Chan et al., 2012). While intramodal sensory gating deficits have been established, the issue of how highly anxious individuals gate sensory information across modalities remains.

In a series of experiments utilizing a crossmodal visual‐tactile sensorimotor attentional selection task, tactile N70 event‐related potential (ERP) amplitude was enhanced when a stimulus was task‐relevant and attenuated when irrelevant (Adams et al., 2017). The N70 (or N67) tactile ERP is a negative peak generated in the primary somatosensory cortex that occurs early in the tactile sensory processing stream (Yamaguchi & Knight, 1990). Importantly, inhibition of the prefrontal cortex with continuous theta burst stimulation (cTBS) reduced N70 amplitude facilitation when attending toward a tactile stimulus relative to when attending away (Adams et al., 2019). In addition to the N70 ERP, the P2 visual ERP was significantly attenuated when participants were instructed to attend away from visual and toward tactile stimuli compared to toward visual stimuli (Adams et al., 2017). However, this effect was variable and was not replicated in later work (Adams et al., 2019). The P2 is a mid‐latency visual ERP generated in the occipital cortex (Adams et al., 2017, 2019). Later ERPs like the P2 are thought to reflect more “integrative cognitive processing” than earlier peaks, though the functional significance of the P2 is under investigation (Crowley & Colrain, 2004). Overall, the role of prefrontal influences in mid‐latency visual processing remains enigmatic. Based on replicated past findings demonstrating attentional sensitivity of the tactile N70 and the PFC's importance in its modulation, this ERP in particular, emerged as a potential marker of attention and task relevance.

The primary objective of the current study was to evaluate potential interaction effects between trait anxiety and task relevance‐based attentional modulation on sensory processing and gating of visual and tactile stimuli. The temporal precision of ERP analysis provided insight into whether and at which time points in the visual and somatosensory processing streams trait anxiety showed a relationship to markers of sensory and cognitive processing. Given past findings (Adams et al., 2017, 2019), it was hypothesized that if high trait anxiety impaired the top‐down control of attention, the tactile N70 ERP amplitude and latency would be modulated by the interaction of trait anxiety and attentional task relevance (attention toward visual or tactile, attention away from visual or tactile), with the null hypothesis being a lack of an interaction. Exploratory analysis of other early somatosensory (P50, P100, and N140) and visual ERPs (P1, N1, P2, and P3) addressed the possibility for other ERPs, particularly those occurring later in the sensory processing stream (e.g., the visual P2 and P3), to reveal potential attentional modulation based on task relevance relative to trait anxiety. More specifically, it was hypothesized that there would be a positive relationship between trait anxiety and distractor ERP amplitude due to reduced top‐down attentional influences related to distractor inhibition, whereas target stimulus processing would be unaffected. The secondary aim of this study was to determine whether trait anxiety influenced sensorimotor performance, as evaluated by distractor cost, when exposed to a stimulus presented concurrently with a distractor compared to without one. Distractor cost represented the relative effectiveness in inhibiting the distractor during the behavioral component of the task. Based on prior findings, it was hypothesized that distractor cost would not relate to trait anxiety due to potential compensatory mechanisms and differences in sensory gating related to trait anxiety.

## 2 MATERIALS AND METHODS

### 2.1 Participants

Sample size was estimated with an a priori power calculation using G*Power 3.1 software (v3.1.9.6; Faul et al., 2009, 2007). Using the “Linear multiple regression: Fixed model, *R* ^2^ deviation from zero” setting in the *F* test family, sample size was estimated with the following specifications: large effect size *f* ^2^ = 0.35, alpha error probability = .05, power = 0.8, and one predictor (trait anxiety score). This calculation output an a priori sample size of 25 participants.

Twenty‐nine healthy young adults were recruited to participate in this study. Exclusion criteria were as follows: neurological illness or impairment, diagnosis of a psychiatric disorder other than generalized anxiety disorder (GAD), a history of brain injury, concussion, or substance abuse, left‐handedness, and psychotropic drug consumption 2 weeks prior. Three subjects were excluded from the analyses: one female (age = 36) was removed due to confusion tied to English as a second language, one male (age = 21) later disclosed an ADHD diagnosis, and one male (age = 24) misunderstood the task. This study was approved by the University of Waterloo Research Ethics Board. All participants provided written informed consent before participation.

Before attending the experimental session, participants completed an online prescreening questionnaire to assess eligibility (Qualtrics, Provo, UT, USA). During the session, a shortened version of the Edinburgh Handedness Inventory (EHI) confirmed right‐handedness (Oldfield, 1971; Veale, 2014). Trait anxiety was evaluated with the State–Trait Anxiety Inventory for Adults™ Form Y2 (STAI‐Y2), a 20‐item measure of self‐reported levels of anxiety based on a 4‐point Likert scale (Spielberger et al., 1983). The state form (STAI‐Y1) was not administered due to the present study's focus on anxiety as a stable personality trait. Participant characteristics of the final sample are presented in Table 1.

**TABLE 1:** Participant characteristics.

	Participant characteristics
*N*	26
Sex	16F, 10M
Age (*M* ± SD, range)	22.69 ± 3.18, 18–33 years
STAI‐Y2 score (*M* ± SD, Mdn)	41.35 ± 10.60, 39

Abbreviations: F, female; M, male. STAI‐Y2, State–Trait Anxiety Inventory trait scale.

### 2.2 Experimental task

**FIGURE 1:**
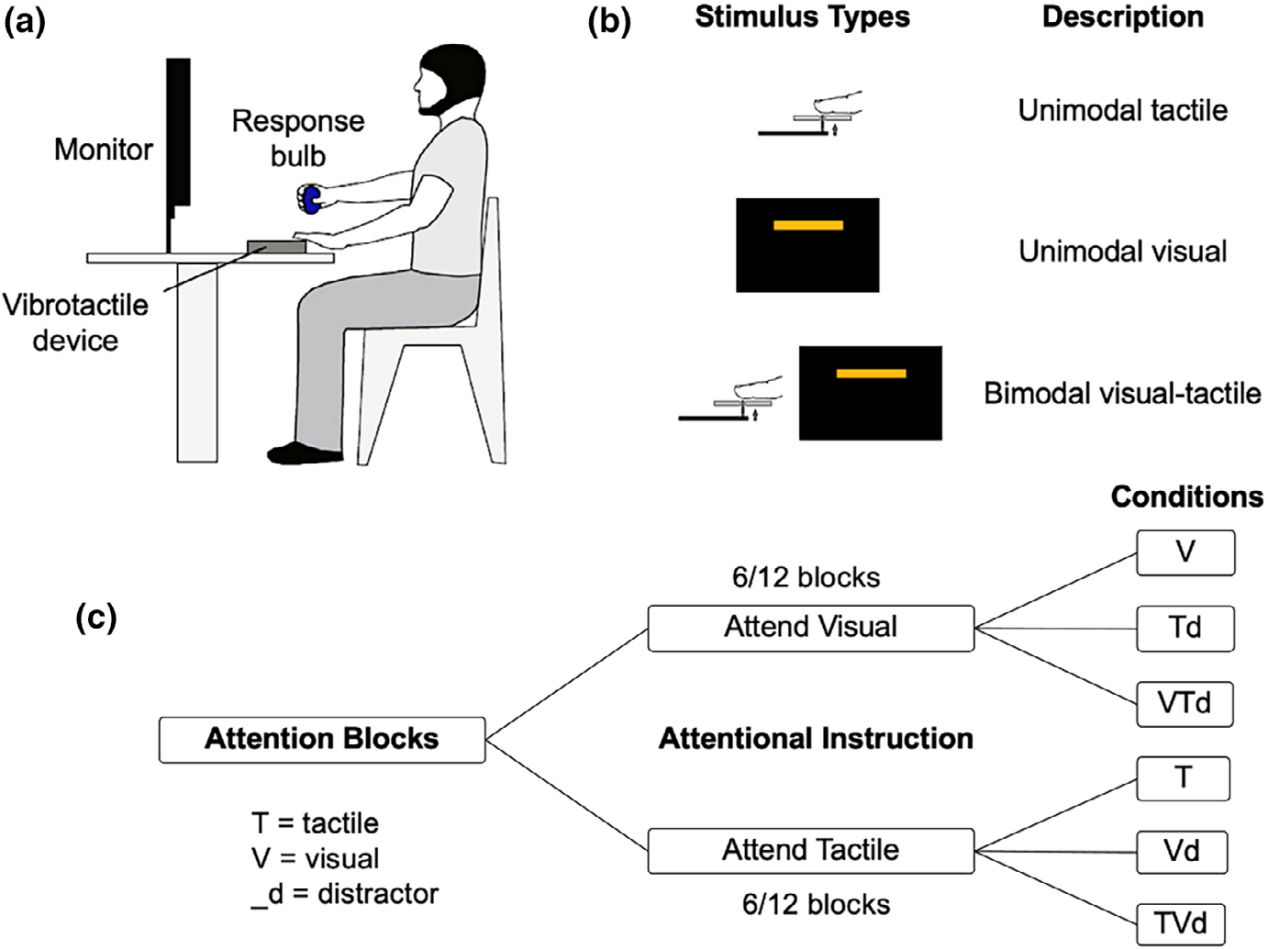
Experimental paradigm. (a) Setup (Adams et al., 2019, 2020). (b) Stimulus types: unimodal tactile, unimodal visual, or bimodal tactile and visual (simultaneous). Tactile stimuli were vibrations of varying intensities to the left index finger from the vibrotactile device. Visual stimuli were orange horizontal bars of varying elevations. (c) Study block types and conditions (12 total, 6 per attentional instruction). “Attend tactile” condition: participants responded to unimodal tactile trials, ignored unimodal visual stimuli, and responded only to tactile while ignoring visual components in bimodal trials. “Attend visual” condition: the opposite was to be performed. Distractor stimulus components are followed by the letter “d” for distractors (e.g., Td, tactile distractor). V: attend visual, unimodal; Td: ignore tactile, unimodal; VTd: attend visual + ignore tactile, bimodal; T: attend tactile, unimodal; Vd: ignore visual, unimodal; TVd: attend tactile + ignore visual, bimodal.

Participants were instructed to fixate their gaze on a computer monitor in front of them which delivered visual stimuli (see Figure 1a). The volar surface of the second digit of their left hand rested on a vibrotactile device that delivered tactile stimuli. The experimental task required participants to respond by applying a corresponding grip force to a pressure‐sensitive hollow rubber bulb that was connected to a pressure sensor with their right hand.

Stimuli were presented unimodally as visual or vibrotactile, or simultaneously (bimodal) (see Figure 1b). A single trial consisted of one stimulus, with a total of 60 trials per experimental block. Each stimulus was presented for 500 ms with 3 s between trials, for a total of 3.5 s per trial. Each block lasted 3.5 min. Twelve experimental blocks contained a total of 720 trials. The experimental task lasted approximately 45 min.

The focus of the visual stimulus was a 6‐cm wide orange bar displayed at the center of a 14.7‐cm high black box on a black background on the monitor. The bar appeared at varying heights within the box (*M* ± SD = 8 ± 4.25 cm; range 1.5–14.5 cm). Tactile stimuli were delivered to the volar surface of the left index finger using a custom vibrotactile device featuring a small modified speaker with an affixed blunt plastic rod protruding vertically through plastic casing. Using a custom‐made program in LabVIEW (version 8.5, National Instruments, Austin, TX), vibrotactile stimuli were created by generating analog vibratory signals from digital waveforms (NI USB‐6341, National Instruments, TX, USA) and amplifying them (Bryston 2BLP, ON, CA). The amplitude of the voltage driving the vibrotactile device led to proportional changes in vibration amplitude on the finger based on Graham et al. (2001). Amplitude remained constant within a trial and varied randomly between trials (driving voltage *M* ± SD = 132 ± 89 mV, range = 26–500 mV). Vibration frequency was fixed at 25 Hz. Amplitudes across trials were randomized, with six randomized stimulus waveforms. Participants wore earbuds delivering white noise during the experiment to prevent auditory perception of the vibrotactile stimuli (White Noise Lite, TMSOFT version 7.8.7, Apple App Store).

Before the experimental blocks, participants completed a 5‐min training session to familiarize themselves with the relationship between the amplitudes of the stimuli and the required grip force to generate a maximally accurate response. Subjects were instructed to squeeze the response bulb with enough force to elevate a blue second horizontal bar to match the height of the visual stimulus (orange bar of variable height). Applying force to the rubber bulb propagated a change in air pressure within a rubber tube, and a pressure sensor recorded this as a voltage proportional to the applied pressure. Pressure sensor calibration ensured that no force applied to the bulb corresponded to 0 mV. The resulting voltage was sampled and digitized at 1000 Hz (NI USB‐6341, National Instruments, TX, USA) and stored in custom software (LabVIEW 8.5, National Instruments, TX, USA). During training, vibrotactile stimuli and their amplitude were controlled by a custom LabVIEW program so that when participants applied force to the response bulb based on the amplitudes of the visual stimuli, a vibration (25 Hz) of proportional strength was applied to the volar surface of their left index finger. The greater the force applied to the bulb, the greater the visual feedback (height of the adjustable blue bar) and tactile feedback (amplitude of vibrotactile feedback to the left index). This form of feedback was only present during the training session to teach the participant the association between different stimulus modalities.

During the experimental session, participants were instructed to attend to a single modality within a given experimental block—visual or tactile. Instructions were counterbalanced between participants and interleaved, with an equal number of blocks for each modality (6 out of 12). In visual attention blocks, participants attended and responded to all visual stimuli (V and visual component of VTd) and ignored tactile stimuli (Td and tactile component of VTd). In tactile attention blocks, participants attended and responded to all tactile stimuli (T and tactile component of TVd) and ignored visual stimuli (Vd and visual component of TVd) (see Figure 1c). Subjects were instructed to respond to the targets by applying the appropriate grip force to the rubber bulb corresponding to the stimulus amplitude of the modality they were instructed to attend toward. Visual and tactile feedback was not provided during experimental blocks.

### 2.3 Data collection and processing

#### 2.3.1 EEG data acquisition

Behavioral data were recorded using a custom LabVIEW program. This program sent event codes to a continuous EEG file indicating the precise stimulus timing and type. Continuous EEG files commenced at the start of each experimental block and ended once all trials of a given block were completed. Trial types (V, T, Vd, Td, VTd, and TVd) were assigned event codes during the experimental task, allowing for ERP analyses time‐locked to stimulus onset.

EEG data were recorded from 11 sites (32‐channel Quik‐Cap, Neuroscan, Compumedics, NC, USA) with Ag/AgCl electrodes in accordance with the 10–20 international system for electrode placement and referenced to linked mastoid electrodes: FP2, FCz, Cz, CP4, C4, P4, CP3, Pz, Oz, O1, and O2. Electrical impedance was maintained at less than 5 kiloOhms (kΩ). EEG data were acquired with a DC‐30 Hz filter, amplified, and digitized at 500 Hz (SynAmps2, Scan 4.5, Compumedics Neuroscan, NC, USA) prior to being saved for offline analysis.

#### 2.3.2 ERP analysis

EEG data analysis occurred offline in MATLAB (MATLAB, 2022) using EEGLAB (Delorme & Makeig, 2004) and ERPLAB (Lopez‐Calderon & Luck, 2014) plugins. A 0.1 Hz high‐pass filter was applied during preprocessing in EEGLAB, for a total bandpass of 0.1–30 Hz. Artifact detection and ERP operations were performed in ERPLAB. Epochs were 600 ms in length, beginning 100 ms prior to stimulus onset and extending to 500 ms following stimulus exposure. Epochs were corrected to the 100 ms prestimulus baseline.

Automatic artifact detection was conducted in ERPLAB using two criteria: (1) simple voltage threshold of −75 to +75 microvolts at FP2 to flag potential blinks and (2) moving window peak‐to‐peak threshold of −65 to +65 microvolts at all electrodes (window full width of 200 ms and window step of 100 ms). Each epoch was manually inspected and trials with noticeable artifacts (e.g., eye blinks and facial muscle flexion) were excluded.

Mean ERP amplitudes and latencies were extracted for each stimulus type based on predetermined latency windows: somatosensory – P50 (45–75 ms), N70 (60–80 ms), P100 (80–120 ms), N140 (125–175 ms); visual – P1 (125–175 ms), N1 (180–220 ms), P2 (225–285 ms) (Adams et al., 2017, 2019, 2020), and P3 (295–500 ms) (Polich, 2007). Based on past work, ERPs were measured from predetermined sets of electrodes (Adams et al., 2017, 2019, 2020). The P50, N70, P100, and N140 somatosensory‐evoked ERPs occur in the cerebral cortex following exposure to a tactile stimulus. The P50 and N70, the earliest tactile ERPs, were recorded from the CP4 electrode overlaying the primary somatosensory cortex (SI) contralateral to stimulation (Hämäläinen et al., 1990; Yamaguchi & Knight, 1990). CP3 was collected to visually inspect the lateralization of the P50/N70. Evoked around the secondary somatosensory cortex (SII) and posterior parietal areas, respectively (Desmedt & Tomberg, 1989), the P100 and N140's neuronal generators are thought to have bilateral receptive fields (Hämäläinen et al., 1990) and project fronto‐centrally (Desmedt & Tomberg, 1989). Thus, both ERPs were recorded from FCz and Cz. To capture visual ERPs, central parietal (Pz) and occipital (Oz, O2, and O1) electrode data were collected. The P1, N1, and P2 visual ERPs are evoked more posteriorly in the occipital lobe which houses the visual cortex (Arroyo et al., 1997; Hillyard & Anllo‐Vento, 1998). The visual target P3 (P3b) occurs in response to a target stimulus and is thought to arise from a combination of cortical and subcortical generators, peaking in parietal areas (reviewed in Herrmann & Knight, 2001; Polich, 2007). From these predetermined electrodes, measurements included in the analysis were derived from the single electrode that showed the largest evoked potential amplitude in the group‐averaged traces (Adams et al., 2017, 2019, 2020).

ERP amplitudes were measured relative to the baseline‐corrected prestimulus period (0–100 ms). In total, 112–to 120 trials were collected for each stimulus type. After eliminating contaminated trials, a mean of 91 artifact‐free epochs per condition were included. On average, 77% of tactile trials and 80% of visual trials were included in the final waveforms.

Participants were excluded from the analysis of the ERP in question if there was no discernable potential in any condition within or around the predetermined time frame. One participant's data were excluded from the P50 and N70 analysis due to noise, one participant did not show tactile P100 and N140 ERPs, another did not show visual P1 or N1 ERPs, and two additional individuals did not show N1 ERPs. A total of 26 participants were included in visual P2 and P3 analysis, 25 in visual P1 and tactile P50, N70, P100, and N140, and 23 in visual N1.

#### 2.3.3 Behavioral analysis

Behavioral data were analyzed by comparing the target stimulus amplitude with the amplitude of the participant's response, generated by squeezing the pressure‐sensitive bulb. Performance was measured by the percentage of the ideal response on a trial‐to‐trial basis. Percentage of the ideal response was calculated as the percent difference in voltage between the response required by the tactile or visual stimulus (driving voltage) and the force exerted (voltage detected by the pressure sensor). Because of the objective of assessing how trait anxiety might impact performance with compared to without a distractor, a distractor cost score was calculated for both sensory modalities (tactile with visual distractors and visual with tactile distractors):

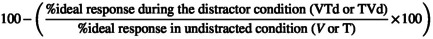




### 2.4 Statistical analysis

Statistical analyses were conducted using R (version 4.3.2; R Core Team, 2021) and RStudio (version 2022.07.1.554; RStudio Team, 2022). Linear mixed models (LMMs) were run using the *lmerTest* package (version 3.1.3; Kuznetsova et al., 2017) to determine the effects of trait anxiety and attention on ERP amplitudes and latencies in response to unimodal (visual or tactile) and bimodal (visual‐tactile) stimuli. Subject was entered as the random factor, with Trait Anxiety (STAI‐Y2 score) as a continuous fixed factor and attention (toward vs. away) as a categorical fixed factor. The significance of the predictors and interactions in the LMM was calculated using *lmerTest*, which estimates degrees of freedom and generates *p*‐values for mixed models using Satterthwaite's method. All analyses were set at a confidence interval of 95%. LMMs were fit by residual maximum likelihood (REML) estimation. Inspection of the residuals using Q–Q plots and scatterplots did not reveal any significant deviations from normality or homoscedasticity. No significant outliers were detected, and all ERP data were included in the analysis. Main effects and interactions were broken down post hoc by *emtrends* and *emmeans* in the emmeans package (version 1.7.5; Lenth, 2022) to extract linear marginal means of linear trends (Trait Anxiety) or to compare between conditions (Attention). *F*‐tests were performed on the LMMs to determine significance (i.e., of the main effects and interactions) and effect sizes. Effect sizes were interpreted based on standardized ANOVA effect size magnitudes: 0.01 ≤ *η*_p_^2^ < 0.06 (small effect), and 0.06 ≤ *η*_p_^2^ < 0.14 (medium effect), 0.14 ≤ *η*_p_^2^ (large effect) (Cohen, 1988).

The LMM procedure was also performed on the subjects' behavioral response data to investigate the potential influences of trait anxiety and distractor sensory modality on performance accuracy. Distractor cost was entered as the dependent variable, Subject was entered as the random factor, Trait Anxiety (STAI‐Y2 score) was added as a continuous fixed factor, and Sensory Modality (crossmodal tactile or visual) was entered as a categorical fixed factor. The *rstatix* package (version 0.7.0; Kassambara, 2012) was used to identify extreme points from each individual's response data (Q1 − 3IQR and Q3 + 3IQR), which, in the rare case they were present (6.15% of trials), were flagged and removed prior to analysis. Only correct responses were included in analysis. Inspection of Q–Q residual plots and residual scatterplots did not reveal any significant deviations from normality or homoscedasticity. The LMMs were fit by REML estimation.

## 3 RESULTS

### 3.1 Event‐related potentials

**FIGURE 2:**
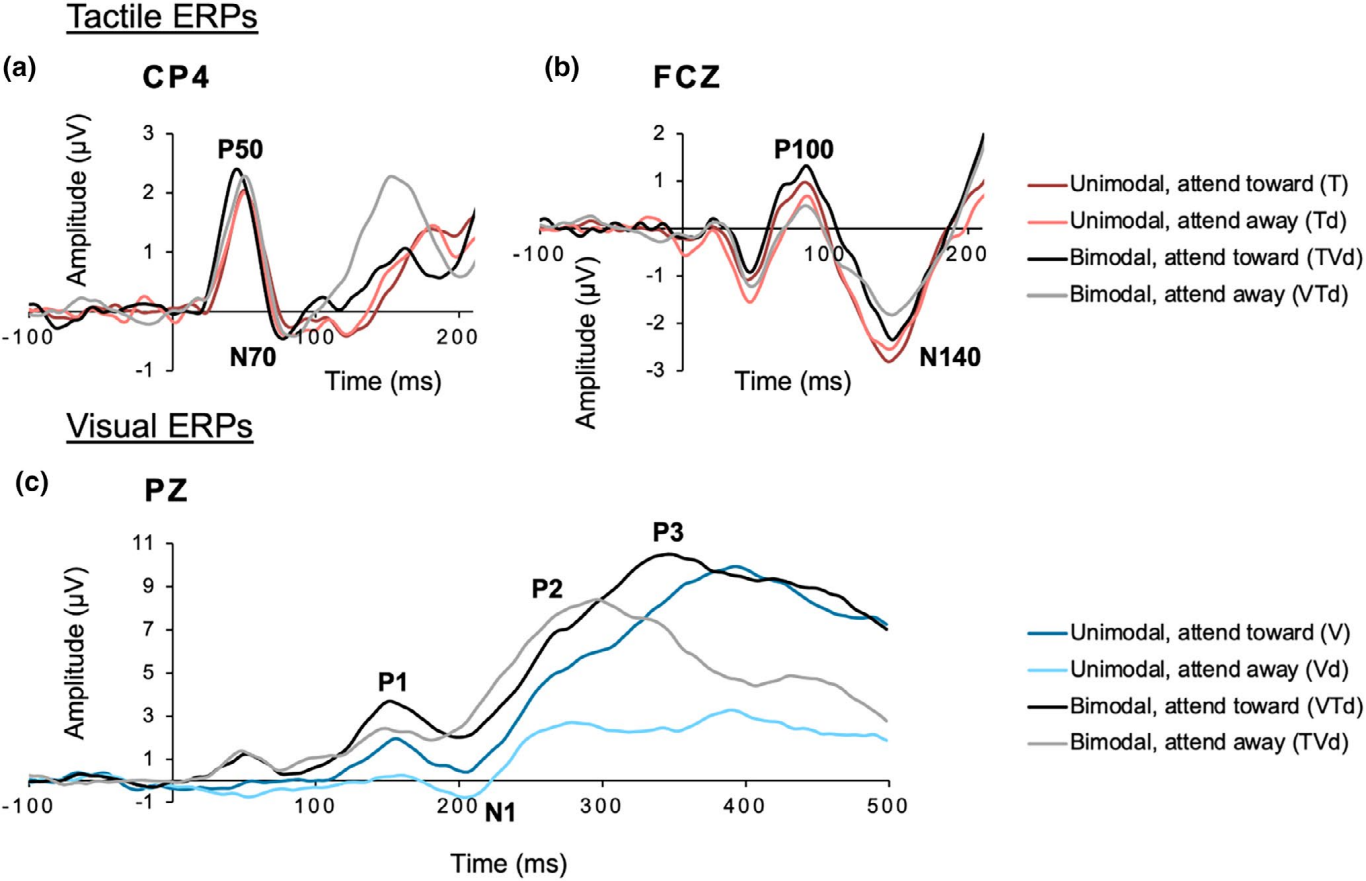
Grand averaged ERP waveforms. The *x*‐axis represents time (ms) relative to stimulus onset. The *y*‐axis represents voltage baseline corrected to the pre‐stimulus period (μV). Tactile ERPs: (a) P50 and N70 tactile ERPs at electrode CP4. (b) P100 and N140 tactile ERPs at electrode CZ. Visual ERPs: (c) visual ERPs at electrode PZ.

Tactile and visual ERPs of interest are demonstrated in grand averages of each condition in Figure 2. Linear mixed analysis with Trait Anxiety and Attention as fixed factors and Subject as the random factor were conducted and to assess whether trait anxiety and attentional modulation impact properties of visual and tactile ERPs when presented unimodally or bimodally.

#### 3.1.1 Tactile ERPs

##### Unimodal stimuli

None of the LMMs on amplitude or latency of the tactile P50, N70, P100, or N140 ERPs yielded any significant outcomes (Table 2).

**TABLE 2:** Statistical results for LMM of trait anxiety and attention on amplitude and latency of tactile ERPs elicited by unimodal tactile stimuli.

	Interaction_Trait Anxiety×Attention_	Main effect_Trait Anxiety_	Main effect_Attention_
Amplitude			
P50	*F*_1,23_ = 0.09, *p* = .77, *η*_p_^2^ = 0.004	*F*_1,23_ = 0.00, *p* = .99, *η*_p_^2^ < 0.001	*F*_1,23_ = 0.00, *p* = .96, *η*_p_^2^ < 0.001
N70	*F*_1,23_ = 0.01, *p* = .92, *η*_p_^2^ < 0.001	*F*_1,23_ = 0.30, *p* = .59, *η*_p_^2^ = 0.01	*F*_1,23_ = 0.11, *p* = .75, *η*_p_^2^ = 0.005
P100	*F*_1,23_ = 0.00, *p* = .99, *η*_p_^2^ < 0.001	*F*_1,23_ = 1.58, *p* = .22, *η*_p_^2^ = 0.06	*F*_1,23_ = 0.10, *p* = .76, *η*_p_^2^ = 0.004
N140	*F*_1,23_ = 0.19, *p* = .67, *η*_p_^2^ = 0.008	*F*_1,23_ = 0.04, *p* = .84, *η*_p_^2^ = 0.002	*F*_1,23_ = 0.48, *p* = .49, *η*_p_^2^ = 0.02
Latency			
P50	*F*_1,22_ = 0.04, *p* = .84, *η*_p_^2^ = 0.002	*F*_1,23_ = 1.29, *p* = .27, *η*_p_^2^ = 0.05	*F*_1,22_ = 0.00, *p* = .98, *η*_p_^2^ < 0.001
N70	*F*_1,23_ = 0.16, *p* = .70, *η*_p_^2^ = 0.007	*F*_1,23_ = 0.02, *p* = .90, *η*_p_^2^ < 0.001	*F*_1,23_ = 0.59, *p* = .45, *η*_p_^2^ = 0.02
P100	*F*_1,22_ = 0.40, *p* = .53, *η*_p_^2^ = 0.02	*F*_1,22_ = 0.57, *p* = .46, *η*_p_^2^ = 0.03	*F*_1,22_ = 0.31, *p* = .58, *η*_p_^2^ = 0.01
N140	*F*_1,23_ = 1.29, *p* = .27, *η*_p_^2^ = 0.05	*F*_1,23_ = 0.09, *p* = .77, *η*_p_^2^ = 0.004	*F*_1,23_ = 1.44, *p* = .24, *η*_p_^2^ = 0.061

##### Bimodal stimuli

None of the LMMs on amplitude or latency of the tactile P50, N70, P100, or N140 ERPs for bimodal visual‐tactile stimuli yielded any significant outcomes (Table 3).

**TABLE 3:** Statistical results for LMM of trait anxiety and attention on amplitude and latency of tactile ERPs for bimodal visual‐tactile stimuli.

	Interaction_Trait Anxiety×Attention_	Main effect_Trait Anxiety_	Main effect_Attention_
Amplitude			
P50	*F*_1,23_ = 0.20, *p* = .66, *η*_p_^2^ = 0.009	*F*_1,23_ = 0.12, *p* = .74, *η*_p_^2^ = 0.005	*F*_1,23_ = 0.29, *p* = .60, *η*_p_^2^ = 0.01
N70	*F*_1,23_ = 0.01, *p* = .94, *η*_p_^2^ = 0.003	*F*_1,23_ = 0.05, *p* = .82, *η*_p_^2^ = 0.002	*F*_1,23_ = 0.04, *p* = .84, *η*_p_^2^ = 0.002
P100	*F*_1,23_ = 1.68, *p* = .21, *η*_p_^2^ = 0.07	*F*_1,23_ = 0.12, *p* = .73, *η*_p_^2^ = 0.005	*F*_1,23_ = 0.83, *p* = .37, *η*_p_^2^ = 0.04
N140	*F*_1,23_ = 0.74, *p* = .40, *η*_p_^2^ = 0.03	*F*_1,23_ = 0.29, *p* = .60, *η*_p_^2^ = 0.01	*F*_1,23_ = 0.83, *p* = .37, *η*_p_^2^ = 0.03
Latency			
P50	*F*_1,23_ = 0.16, *p* = .69, *η*_p_^2^ = 0.007	*F*_1,23_ = 0.61, *p* = .44, *η*_p_^2^ = 0.03	*F*_1,23_ = 0.14, *p* = .71, *η*_p_^2^ = 0.006
N70	*F*_1,23_ = 1.51, *p* = .23, *η*_p_^2^ = 0.06	*F*_1,23_ = 0.07, *p* = .79, *η*_p_^2^ = 0.003	*F*_1,23_ = 2.08, *p* = .16, *η*_p_^2^ = 0.08
P100	*F*_1,22_ = 2.04, *p* = .17, *η*_p_^2^ = 0.08	*F*_1,22_ = 0.02, *p* = .89, *η*_p_^2^ < 0.001	*F*_1,22_ = 2.58, *p* = .12, *η*_p_^2^ = 0.11
N140	*F*_1,23_ = 0.03, *p* = .86, *η*_p_^2^ = 0.001	*F*_1,23_ = 0.39, *p* = .54, *η*_p_^2^ = 0.02	*F*_1,23_ = 0.07, *p* = .79, *η*_p_^2^ = 0.003

#### 3.1.2 Visual ERPs

##### Unimodal stimuli

**FIGURE 3:**
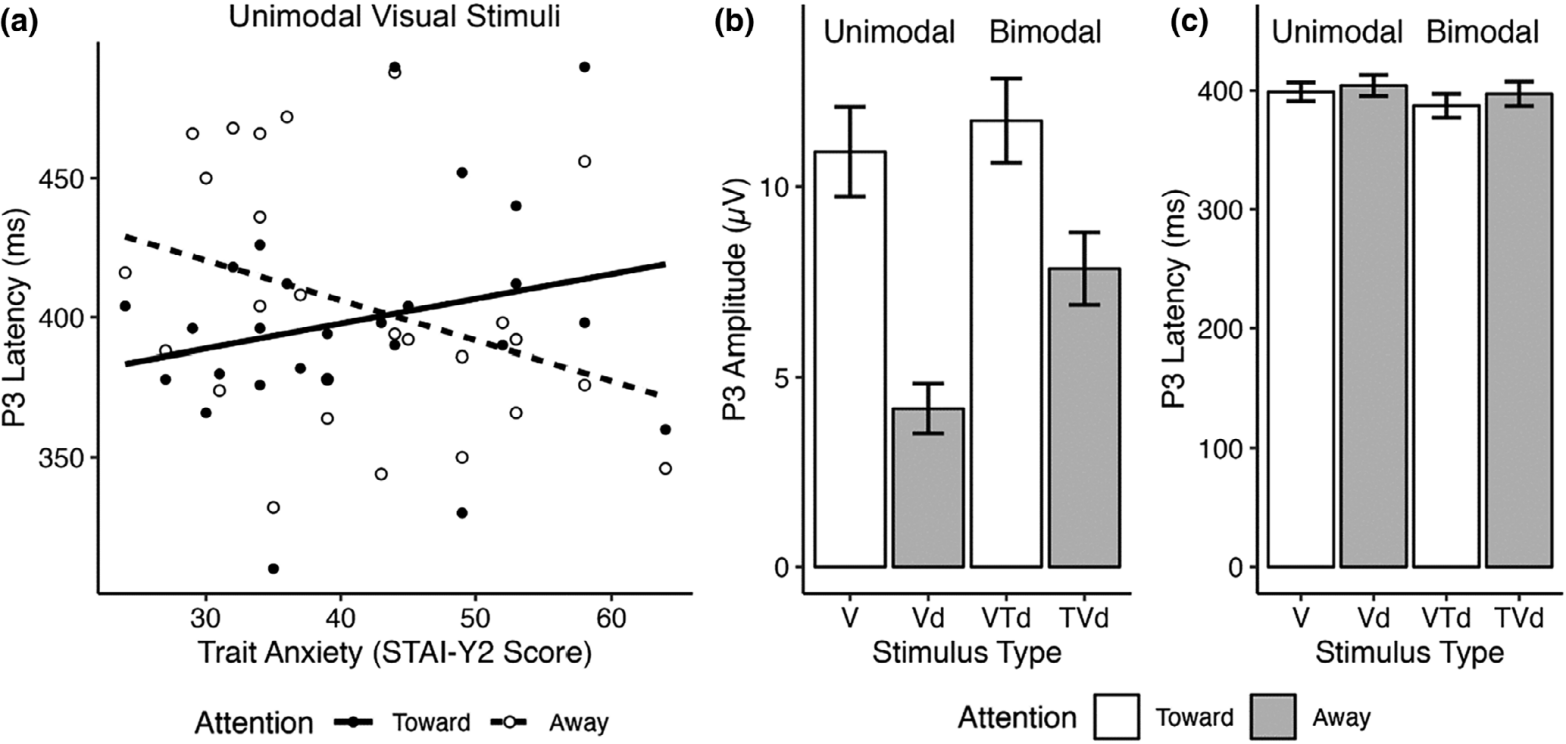
Visual P3 amplitude and latency results from electrode Pz. (a) LMM analysis found a trait anxiety and attention interaction of visual P3 ERP latencies in response to unimodal visual stimuli. (b) P3 amplitudes for each visual condition. A significant main effect of attention on P3 amplitude in response to bimodal stimuli was detected. (c) P3 latencies for each visual condition.

The LMM for visual P3 latency in response to unimodal visual stimuli revealed a large Trait Anxiety x Attention interaction (*F*_1,24_ = 9.82, *p* = .005, *η*_p_^2^ = 0.29) and a large main effect of Attention (*F*_1,24_ = 10.29, *p* = .004, *η*_p_^2^ = 0.30) (Table 4). The significant interaction was driven by increasingly longer P3 latencies as trait anxiety score increased when attending toward the unimodal visual stimulus. In contrast, P3 latency decreased as trait anxiety score increased when attending away from the unimodal visual stimulus (Figure 3a). None of the other LMM for unimodal visual stimuli for the P1, N1, P2, or visual P3 amplitude yielded significant effects.

**TABLE 4:** Statistical results for LMM of trait anxiety and attention on amplitude and latency of tactile ERPs elicited by unimodal visual stimuli.

	Interaction_Trait Anxiety×Attention_	Main effect_Trait Anxiety_	Main effect_Attention_
Amplitude			
P1	*F*_1,23_ = 0.01, *p* = .93, *η*_p_^2^ < 0.001	*F*_1,23_ = 0.01, *p* = .93, *η*_p_^2^ < 0.001	*F*_1,23_ = 0.41, *p* = .53, *η*_p_^2^ = 0.02
N1	*F*_1,21_ = 0.72, *p* = .40, *η*_p_^2^ = 0.03	*F*_1,21_ = 2.32, *p* = .14, *η*_p_^2^ = 0.10	*F*_1,21_ = 1.12, *p* = .30, *η*_p_^2^ = 0.05
P2	*F*_1,24_ = 0.08, *p* = .78, *η*_p_^2^ = 0.003	*F*_1,24_ = 0.00, *p* = 1.00, *η*_p_^2^ < 0.001	*F*_1,24_ = 1.06, *p* = .31, *η*_p_^2^ = 0.04
P3	*F*_1,24_ = 0.13, *p* = .72, *η*_p_^2^ = 0.005	*F*_1,24_ = 0.06, *p* = .80, *η*_p_^2^ = 0.003	*F*_1,24_ = 1.40, *p* = .25, *η*_p_^2^ = 0.05
Latency			
P1	*F*_1,23_ = 0.65, *p* = .43, *η*_p_^2^ = 0.03	*F*_1,23_ = 1.08, *p* = .31, *η*_p_^2^ = 0.04	*F*_1,23_ = 0.48, *p* = .49, *η*_p_^2^ = 0.02
N1	*F*_1,21_ = 3.45, *p* = .08, *η*_p_^2^ = 0.14	*F*_1,21_ = 0.55, *p* = .47, *η*_p_^2^ = 0.03	*F*_1,21_ = 2.03, *p* = .17, *η*_p_^2^ = 0.09
P2	*F*_1,24_ = 0.43, *p* = .52, *η*_p_^2^ = 0.02	*F*_1,24_ = 2.85, *p* = .10, *η*_p_^2^ = 0.11	*F*_1,24_ = 0.50, *p* = .48, *η*_p_^2^ = 0.02
P3	***F***_**1,24**_ **= 9.82, *p* = .005, *η***_**p**_^**2**^ **= 0.29**	*F*_1,24_ = 0.16, *p* = .70, *η*_p_^2^ = 0.006	***F***_**1,24**_ **= 10.29, *p* = .004, *η***_**p**_^**2**^ **= 0.30**

*Note*: Significant effects are shown in bold.

##### Bimodal stimuli

The LMM for visual P3 amplitude in response to bimodal visual‐tactile stimuli revealed a large main effect of Attention (*F*_1,24_ = 5.34, *p* = .03, *η*_p_^2^ = 0.18), where visual P3 amplitude was greater when attending toward the visual component of the bimodal stimulus compared to when attending away (Table 5, Figure 3b). The LMM on visual P3 latency did not yield any significant outcomes. Bar graphs representing mean P3 latency are presented in Figure 3c. Furthermore, the LMM for visual N1 amplitude for bimodal visual‐tactile stimuli indicated a large, significant main effect of Trait Anxiety (*F*_1,21_ = 4.39, *p* = .048, *η*_p_^2^ = 0.17), which showed a positive relationship between N1 amplitude and Trait Anxiety. The LMM on visual N1 latency did not yield any significant effects. Amplitude and latency analyses on the visual P1 and P2 ERPs also did not indicate any significant effects (Table 5).

**TABLE 5:** Statistical results for LMM of trait anxiety and attention on amplitude and latency of visual ERPs for bimodal visual‐tactile stimuli.

	Interaction_Trait Anxiety×Attention_	Main effect_Trait Anxiety_	Main effect_Attention_
Amplitude			
P1	*F*_1,23_ = 0.10, *p* = .75, *η*_p_^2^ = 0.004	*F*_1,23_ = 1.10, *p* = .31, *η*_p_^2^ = 0.05	*F*_1,23_ = 0.57, *p* = .46, *η*_p_^2^ = 0.02
N1	*F*_1,21_ = 3.30, *p* = .08, *η*_p_^2^ = 0.14	***F***_**1,21**_ **= 4.39, *p* = .048, *η***_**p**_^**2**^ **= 0.17**	*F*_1,21_ = 1.80, *p* = .19, *η*_p_^2^ = 0.08
P2	*F*_1,24_ = 0.03, *p* = .85, *η*_p_^2^ = 0.08	*F*_1,24_ = 1.33, *p* = .26, *η*_p_^2^ = 0.02	*F*_1,24_ = 0.08, *p* = .78, *η*_p_^2^ = 0.03
P3	*F*_1,24_ = 0.33, *p* = .57, *η*_p_^2^ = 0.01	*F*_1,24_ = 1.50, *p* = .23, *η*_p_^2^ = 0.06	***F***_**1,24**_ **= 5.34, *p* = .03, *η***_**p**_^**2**^ **= 0.18**
Latency			
P1	*F*_1,23_ = 0.65, *p* = .43, *η*_p_^2^ = 0.03	*F*_1,23_ = 0.29, *p* = .60, *η*_p_^2^ = 0.01	*F*_1,23_ = 0.44, *p* = .51, *η*_p_^2^ = 0.02
N1	*F*_1,21_ = 0.09, *p* = .76, *η*_p_^2^ = 0.004	*F*_1,21_ = 0.03, *p* = .86, *η*_p_^2^ = 0.001	*F*_1,21_ = 0.00, *p* = .96, *η*_p_^2^ < 0.001
P2	*F*_1,24_ = 0.67, *p* = .42, *η*_p_^2^ = 0.03	*F*_1,24_ = 0.01, *p* = .91, *η*_p_^2^ < 0.001	*F*_1,24_ = 0.00, *p* = .96, *η*_p_^2^ < 0.001
P3	*F*_1,24_ = 0.74, *p* = .40, *η*_p_^2^ = 0.03	*F*_1,24_ = 0.10, *p* = .76, *η*_p_^2^ = 0.004	*F*_1,24_ = 1.19, *p* = .29, *η*_p_^2^ = 0.05

*Note*: Significant effects are shown in bold.

### 3.2 Distractor cost to behavioral accuracy

**FIGURE 4:**
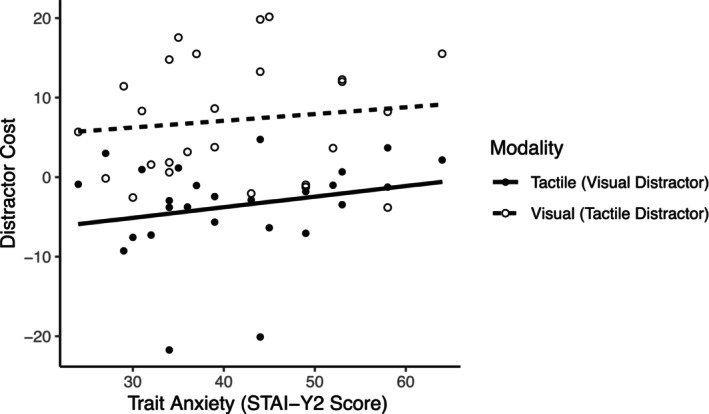
No significant relationship was found between trait anxiety and sensory modality on distractor cost.

Linear mixed analysis with Trait Anxiety and Sensory Modality as fixed factors and Subject as the random factor were conducted to assess whether trait anxiety and sensory modality of the stimulus and crossmodal distractor impact distractor cost to behavioral performance accuracy. There were no significant effects of trait anxiety and sensory modality on distractor cost (Interaction_Trait AnxietyxSensory Modality_: *F*_1,48_ = 0.07, *p* = .79, *η*_p_^2^ = 0.001; Main effect_Trait Anxiety_: *F*_1,48_ = 1.42, *p* = .24, *η*_p_^2^ = 0.03; Main effect_Sensory Modality_: *F*_1,48_ = 2.70, *p* = .11, *η*_p_^2^ = 0.05) (Figure 4).

## 4 DISCUSSION

While past work has focused largely on visual processing, the current study examined the relationship between trait anxiety and sensorimotor processing of visual and tactile stimuli with and without crossmodal distractors. The primary hypothesis was that ERP properties, particularly those of the tactile N70, would relate to the interaction of trait anxiety and attentional task relevance. Tactile ERPs (P50, N70, P100, and N140) were not modulated by trait anxiety regardless of whether they were presented alone or with a visual stimulus. Out of the visual ERPs tested (P1, N1, P2, and P3), the P3 latency showed a significant interaction, where a large and significant interaction between trait anxiety and attention for visual P3 latency for unimodal visual stimuli was detected. There was also a large main effect of attention on visual P3 amplitude in response to bimodal visual‐tactile stimuli, where visual P3 amplitude was greater when attending toward the visual component of the bimodal stimulus compared to when attending away. Visual N1 amplitude showed a positive relationship with trait anxiety with a large effect size. No other significant ERP‐related effects between trait anxiety and attention were observed. As hypothesized, trait anxiety did not significantly influence behavioral distractor cost of either stimulus type (visual or tactile distractors). Overall, these results suggest that the effects of trait anxiety on visual processing relate to processing delays as opposed to distractor cost on sensorimotor accuracy.

### 4.1 Trait anxiety and attention influence visual P3 latency without a crossmodal distractor

The P3 is a large, broad potential that occurs in response to sensory stimuli of all types (i.e., visual, auditory, and somatosensory) (Dreo et al., 2017). While customarily examined in the context of stimulus predictability and categorization (i.e., in the oddball paradigm), the P3 ERP is also modulated by motivational relevance (see review by Nieuwenhuis et al., 2005). In the current study, while there were no trait anxiety‐related effects on P3 amplitude, there was a strong interaction between trait anxiety and attention on P3 latency in response to unimodal visual stimuli. Trait anxiety showed a positive relationship with stimulus evaluation time when attending toward a task‐relevant unimodal visual stimulus. When exposed to a task irrelevant unimodal visual stimulus, this revealed a negative relationship. Top‐down attentional influences can affect the magnitude and processing speed of ERPs, which is relevant in the case of competing sensory inputs (Gazzaley et al., 2005). Faster latency reflects more efficient processing and facilitation of relevant information extraction (Gazzaley et al., 2005). Furthermore, Corbetta and Shulman (2002) described that a “salience” map, maintained by the dorsal frontoparietal system, combines afferent information with higher cortical influences during visual search. Consistent with Corbetta and Shulman's (2002) and Gazzaley et al.'s (2005) postulations, lower trait anxious individuals may have used this salience map more effectively, with relevant visual information being processed faster than irrelevant information. In comparison, those with high trait anxiety showed the reverse, processing irrelevant visual information more quickly and efficiently than less anxious individuals but longer to process relevant visual information. This finding can be paralleled with anxiety‐related hypervigilance which has been shown in numerous works to cause attentional biases with evolutionary roots (reviewed by Cisler & Koster, 2010), potentially also causing impaired attentional disengagement from threat (Eysenck et al., 2007) and distractor inhibition (see review by Derakshan & Eysenck, 2009) in situations lacking emotionally valent stimuli or induced stress.

The P3 ERP is thought to be linked to the LC‐NE (locus coeruleus‐norephinephrine) system, which is involved in many functions including arousal and attention facilitation (Nieuwenhuis et al., 2005; Ross & Van Bockstaele, 2021). In the LC‐NE system, norepinephrine (NE) impacts cortical and subcortical activity via the locus coeruleus, a collection of NE neuronal cell bodies (Aston‐Jones & Cohen, 2005). The thalamus and hippocampus are thought to be involved in P3 generation (Herrmann & Knight, 2001)—both structures are recipients of projections from the LC, as well as the neocortex (including the PFC) and many other forebrain areas (Bouret & Sara, 2005). Reviewed by Nieuwenhuis et al. (2005), the P3 was proposed to reflect the phasic enhancement of gain as a result of enhanced LC‐NE activity. Thus, when high trait anxious individuals are preparing to respond to a relevant visual stimulus, there may be greater gain in LC‐NE activity and higher cortical influences during sensory processing. However, when instructed to attend to a crossmodal stimulus, these resources may be recruited for the opposite modality, reflecting a slowness in processing of the unimodal visual stimulus when presented as a distractor.

Delayed P3 latencies for task‐relevant visual stimuli are associated with conditions involving diminished prefrontal activation; specifically, major depression (Vandoolaeghe et al., 1998) and post inhibitory cTBS to the left DLPFC (Pinto et al., 2021). In addition, the conclusion that the left DLPFC is hypoactive relative to trait anxiety in situations with low and not high perceptual loads (Bishop, 2009) can potentially explain why this effect was observed in the unimodal visual condition (low perceptual load) but not in the bimodal condition (higher perceptual load). Thus, the longer latencies for task‐relevant visual stimulus processing with higher trait anxiety may relate to diminished prefrontal activation during task‐relevant visual stimulus processing.

### 4.2 N1 amplitude is modulated by trait anxiety for bimodal visual‐tactile stimuli

In the present study, the large, significant main effect of trait anxiety on visual N1 amplitude for bimodal stimuli was detected. A negative neural potential that coincides with the N1 and P2 ERPs is called the processing negativity (PN) (Näätänen, 1982) or “Nd” (Hansen & Hillyard, 1980), which is enhanced when attending toward stimuli. Crowley and Colrain (2004) reasoned that an enhanced N1 is linked to increased attentiveness of the subject to the stimulus due to a summation of N1 and PN/Nd potentials. Thus, indicating a positive relationship between trait anxiety and N1 amplitude may be explained as a possible gradual attentional enhancement toward the visual component of the bimodal stimuli corresponding with higher trait anxiety.

The Trait Anxiety x Attention interaction for N1 amplitude for bimodal stimuli in the present study was large (*η*_p_^2^ = 0.14), suggesting a potentially noteworthy effect that may not reached significance three individuals not showing N1 peaks. Xiu et al. (2022) investigated the relationship between the N1 and P2 ERPs and neuroticsm in a two‐back working memory task, a predisposition to feeling anxious, which has been closely linked to trait anxiety. No between‐group differences in the N1 amplitude or latency between high and low neuroticism groups were detected (Xiu et al., 2022). Other research linking the visual N1 ERP to forms of anxiety is scarce. Further work should explore the relationship between personality traits and markers evoked in pre‐attentional stages of perception to elucidate possible structural and functional neural differences.

### 4.3 The tactile N70 ERP and trait anxiety

In the present study, it was predicted that the effect of higher trait anxiety on ERP amplitudes would parallel that of downregulated PFC activity. This hypothesis was based on Adams et al.'s (2019) findings, which showed that temporary inhibition of the PFC with cTBS impacted the attentional modulation of the tactile N70 ERP. Furthermore, their findings indicated that the prefrontal cortex contributes to the sensory gating of information, wherein inhibition of the PFC with cTBS prevented a facilitation of N70 amplitude in response to attended tactile information that was observed pre‐cTBS (Adams et al., 2019). In the current study, the lack of facilitation of N70 amplitude in response to relevant tactile stimuli was not just observed with higher anxiety, but across all participants. This discrepancy between studies is likely due to the differences in targeted samples (i.e., healthy individuals in the general population vs. individuals with varying levels of anxiety in the current study). Furthermore, unlike the findings of Adams et al. (2017), there was no significant effect of attention on N70 amplitude when attending toward tactile compared to when attending toward the visual modality. The introduction of increased variability in statistical modeling due to the addition of anxiety as a variable may have contributed to the decreased ability to detect significant attention effects compared to the prior study. The small‐to‐medium effects of trait anxiety and attention on this ERP (all small except moderate but insignificant effects detected for the interaction and main effect of attention for bimodal latency) substantiate that trait anxiety has limited effects on tactile somatosensory processing.

### 4.4 Trait anxiety shows no relationship to behavioral distractor cost

In accordance with ACT's notion that anxiety affects performance effectiveness less so than processing efficiency, behavioral results in this study showed that the influence of trait anxiety on distractor cost was characterized by weak estimates of effect size (Trait Anxiety × Sensory Modality interaction *η*_p_^2^ = 0.001, Trait Anxiety main effect *η*_p_^2^ = 0.03). Because the task instructions emphasized accuracy and not reaction time, the task may not have been sufficiently demanding to show a pronounced effect of anxiety on performance.

### 4.5 Additional considerations and limitations

Independent of considering significance, there is evidence of weaker effects with medium‐to‐large effect sizes that could have potential future clinical research applications. For tactile ERPs, some results showed *η*_p_^2^ values of 0.06–0.08, which would be equivalent to moderate effect sizes of practical significance. Possibly noteworthy was the large interaction effect size of Trait Anxiety x Attention on N1 latency for unimodal visual stimuli (*η*_p_^2^ = 0.14), which was caused by a more negative relationship between N1 latency and trait anxiety when attending away from the stimulus compared to toward it. Also potentially worth noting was a moderate main effect size of Trait Anxiety on P2 latency for unimodal visual stimuli (*η*_p_^2^ = 0.11), which was caused by a negative relationship between P2 latency and trait anxiety. The Trait Anxiety x Attention interaction for N1 amplitude was likely weaker due to some individuals not showing N1 peaks, making it thus not as well powered as the other ERPs. However, with the exploratory nature of this study and an overall sample size of 26 individuals, effect sizes to target the most relevant ERPs to highlight an interaction between trait anxiety and attention were obtained.

## 5 CONCLUSIONS

While trait anxiety has been associated with impaired inhibition of irrelevant information, this had largely been documented in a single stimulus modality (usually visual) and had rarely been explored in a bimodal context. The current study's findings demonstrated (1) alterations in electrophysiological correlates of neural processing modulated by the interaction of trait anxiety and attentional relevance as demonstrated in the visual P3 latency for unimodal visual stimuli, but not for bimodal stimuli, (2) no significant interactions for earlier visual or tactile ERPs, and (3) no impact of trait anxiety and sensory modality on distractor cost to behavioral accuracy in the sensorimotor task. The presence of the visual P3 latency Trait Anxiety x Attention interaction for unimodal visual stimuli indicates that there may be alterations in cortical processing related to higher anxiety that did not manifest behaviourally due to the involvement of compensatory neural mechanisms. Future work should address the relationship between trait anxiety, attention, and markers of sensory processing in experimental tasks with higher sensory load and in more ecologically valid situations to increase generalizability to everyday cognition.

## AUTHOR CONTRIBUTIONS

**Michelle V. Faerman:** Conceptualization; formal analysis; investigation; writing – original draft; writing – review and editing. **Kaylena A. Ehgoetz Martens:** Conceptualization; methodology; project administration; supervision; writing – review and editing. **Sean K. Meehan:** Methodology; writing – review and editing. **W. Richard Staines:** Conceptualization; funding acquisition; methodology; project administration; resources; supervision; writing – review and editing.

## FUNDING INFORMATION

Government of Canada Natural Sciences and Engineering Research Council of Canada.

## CONFLICT OF INTEREST STATEMENT

The authors have no conflict of interest to declare.

## Data Availability

Data will be made available upon request.

## References

[psyp14706-bib-0001] Adams, M. S. , Andrew, D. , & Staines, W. R. (2019). The contribution of the prefrontal cortex to relevancy‐based gating of visual and tactile stimuli. Experimental Brain Research, 237(10), 2747–2759. 10.1007/s00221-019-05633-9 31435693

[psyp14706-bib-0002] Adams, M. S. , Niechwiej‐Szwedo, E. , McIlroy, W. E. , & Staines, W. R. (2020). A history of concussion affects relevancy‐based modulation of cortical responses to tactile stimuli. Frontiers in Integrative Neuroscience, 14, 33. 10.3389/fnint.2020.00033 32719591 PMC7350857

[psyp14706-bib-0003] Adams, M. S. , Popovich, C. , & Staines, W. R. (2017). Gating at early cortical processing stages is associated with changes in behavioural performance on a sensory conflict task. Behavioural Brain Research, 317, 179–187. 10.1016/j.bbr.2016.09.037 27641325

[psyp14706-bib-0004] Ansari, T. L. , & Derakshan, N. (2011a). The neural correlates of impaired inhibitory control in anxiety. Neuropsychologia, 49(5), 1146–1153. 10.1016/J.NEUROPSYCHOLOGIA.2011.01.019 21241717

[psyp14706-bib-0005] Ansari, T. L. , & Derakshan, N. (2011b). The neural correlates of cognitive effort in anxiety: Effects on processing efficiency. Biological Psychology, 86(3), 337–348. 10.1016/j.biopsycho.2010.12.013 21277934

[psyp14706-bib-0006] Arroyo, S. , Lesser, R. P. , Poon, W.‐T. , Webber, W. R. S. , & Gordon, B. (1997). Neuronal generators of visual evoked potentials in humans: Visual processing in the human cortex. Epilepsia, 38(5), 600–610. 10.1111/J.1528-1157.1997.TB01146.X 9184607

[psyp14706-bib-0007] Aston‐Jones, G. , & Cohen, J. D. (2005). An integrative theory of locus coeruleus‐norepinephrine function: Adaptive gain and optimal performance. Annual Review of Neuroscience, 28, 403–450. 10.1146/annurev.neuro.28.061604.135709 16022602

[psyp14706-bib-0008] Basten, U. , Stelzel, C. , & Fiebach, C. J. (2011). Trait anxiety modulates the neural efficiency of inhibitory control. Journal of Cognitive Neuroscience, 23(10), 3132–3145. 10.1162/JOCN_A_00003 21391763

[psyp14706-bib-0009] Basten, U. , Stelzel, C. , & Fiebach, C. J. (2012). Trait anxiety and the neural efficiency of manipulation in working memory. Cognitive, Affective, & Behavioral Neuroscience, 12(3), 571–588. 10.3758/S13415-012-0100-3 PMC3400031 22644759

[psyp14706-bib-0010] Berggren, N. , & Derakshan, N. (2012). Attentional control deficits in trait anxiety: Why you see them and why you don't. Biological Psychology, 92(3), 440–446. 10.1016/j.biopsycho.2012.03.007 22465045

[psyp14706-bib-0011] Bishop, S. J. (2009). Trait anxiety and impoverished prefrontal control of attention. Nature Neuroscience, 12(1), 92–98. 10.1038/nn.2242 19079249

[psyp14706-bib-0012] Bouret, S. , & Sara, S. J. (2005). Network reset: A simplified overarching theory of locus coeruleus noradrenaline function. Trends in Neurosciences, 28(11), 574–582. 10.1016/j.tins.2005.09.002 16165227

[psyp14706-bib-0013] Chan, P.‐Y. S. , von Leupoldt, A. , Bradley, M. M. , Lang, P. J. , & Davenport, P. W. (2012). The effect of anxiety on respiratory sensory gating measured by respiratory‐related evoked potentials. Biological Psychology, 91(2), 185–189. 10.1016/J.BIOPSYCHO.2012.07.001 22781313 PMC3612944

[psyp14706-bib-0014] Chao, L. L. , & Knight, R. T. (1995). Human prefrontal lesions increase distractibility to irrelevant sensory inputs. Neuroreport, 6(12), 1605–1610. 10.1097/00001756-199508000-00005 8527724

[psyp14706-bib-0015] Cisler, J. M. , & Koster, E. H. W. (2010). Mechanisms of attentional biases towards threat in anxiety disorders: An integrative review. Clinical Psychology Review, 30(2), 203–216. 10.1016/j.cpr.2009.11.003 20005616 PMC2814889

[psyp14706-bib-0016] Cohen, J. (1988). Statistical power analysis for the behavioural sciences (2nd ed.). L. Erlbaum Associates.

[psyp14706-bib-0017] Corbetta, M. , & Shulman, G. L. (2002). Control of goal‐directed and stimulus‐driven attention in the brain. Nature Reviews Neuroscience, 3(3), 201–215. 10.1038/nrn755 11994752

[psyp14706-bib-0018] Cromwell, H. C. , Mears, R. P. , Wan, L. , & Boutros, N. N. (2008). Sensory gating: A translational effort from basic to clinical science. Clinical EEG and Neuroscience, 39(2), 69–72. 10.1177/155005940803900209 18450171 PMC4127047

[psyp14706-bib-0019] Crowley, K. E. , & Colrain, I. M. (2004). A review of the evidence for P2 being an independent component process: Age, sleep and modality. Clinical Neurophysiology, 115(4), 732–744. 10.1016/j.clinph.2003.11.021 15003751

[psyp14706-bib-0020] Delorme, A. , & Makeig, S. (2004). EEGLAB: An open source toolbox for analysis of single‐trial EEG dynamics including independent component analysis. Journal of Neuroscience Methods, 134, 9–21. http://www.sccn.ucsd.edu/eeglab/ 15102499 10.1016/j.jneumeth.2003.10.009

[psyp14706-bib-0021] Derakshan, N. , & Eysenck, M. W. (2009). Anxiety, processing efficiency, and cognitive performance: New developments from attentional control theory. European Psychologist, 14(2), 168–176. 10.1027/1016-9040.14.2.168

[psyp14706-bib-0022] Desmedt, J. E. , & Tomberg, C. (1989). Mapping early somatosensory evoked potentials in selective attention: Critical evaluation of control conditions used for titrating by difference the cognitive P30, P40, P100 and N140. Electroencephalography and Clinical Neurophysiology/Evoked Potentials Section, 74(5), 321–346. 10.1016/0168-5597(89)90001-4 2476292

[psyp14706-bib-0023] Dreo, J. , Attia, D. , Pirtošek, Z. , & Repovš, G. (2017). The P3 cognitive ERP has at least some sensory modality‐specific generators: Evidence from high‐resolution EEG. Psychophysiology, 54(3), 416–428. 10.1111/PSYP.12800 28039922

[psyp14706-bib-0024] Duley, A. R. , Hillman, C. H. , Coombes, S. , & Janelle, C. M. (2007). Sensorimotor gating and anxiety: Prepulse inhibition following acute exercise. International Journal of Psychophysiology, 64(2), 157–164. 10.1016/J.IJPSYCHO.2007.01.006 17350126

[psyp14706-bib-0025] Eysenck, M. W. , & Derakshan, N. (2011). New perspectives in attentional control theory. Personality and Individual Differences, 50(7), 955–960. 10.1016/J.PAID.2010.08.019

[psyp14706-bib-0026] Eysenck, M. W. , Derakshan, N. , Santos, R. , & Calvo, M. G. (2007). Anxiety and cognitive performance: Attentional control theory. Emotion, 7(2), 336–353. 10.1037/1528-3542.7.2.336 17516812

[psyp14706-bib-0027] Faul, F. , Erdfelder, E. , Buchner, A. , & Lang, A. G. (2009). Statistical power analyses using G*power 3.1: Tests for correlation and regression analyses. Behavior Research Methods, 41(4), 1149–1160. 10.3758/BRM.41.4.1149 19897823

[psyp14706-bib-0028] Faul, F. , Erdfelder, E. , Lang, A.‐G. , & Buchner, A. (2007). G*power 3: A flexible statistical power analysis program for the social, behavioral, and biomedical sciences. Behavior Research Methods, 39(2), 175–191. 10.3758/BF03193146 17695343

[psyp14706-bib-0029] Forster, S. , Elizalde, A. O. N. , Castle, E. , & Bishop, S. J. (2015). Unraveling the anxious mind: Anxiety, worry, and frontal engagement in sustained attention versus off‐task processing. Cerebral Cortex, 25(3), 609–618. 10.1093/cercor/bht248 24062316 PMC4318530

[psyp14706-bib-0030] Gazzaley, A. , Cooney, J. W. , McEvoy, K. , Knight, R. T. , & D'Esposito, M. (2005). Top‐down enhancement and suppression of the magnitude and speed of neural activity. Journal of Cognitive Neuroscience, 17(3), 507–517. 10.1162/0898929053279522 15814009

[psyp14706-bib-0031] Graham, S. J. , Staines, W. R. , Nelson, A. , Plewes, D. B. , & McIlroy, W. E. (2001). New devices to deliver somatosensory stimuli during functional MRI. Magnetic Resonance in Medicine, 46(3), 436–442. 10.1002/mrm.1211 11550233

[psyp14706-bib-0032] Grunwald, T. , Boutros, N. N. , Pezer, N. , von Oertzen, J. , Fernández, G. , Schaller, C. , & Elger, C. E. (2003). Neuronal substrates of sensory gating within the human brain. Biological Psychiatry, 53(6), 511–519. 10.1016/S0006-3223(02)01673-6 12644356

[psyp14706-bib-0033] Hainaut, J.‐P. , & Bolmont, B. (2013). Sensory modalities processing in sensorimotor tasks depends on state and trait anxiety. In M. Voisin & R. Brunel (Eds.), New developments in sensory processing research (pp. 83–108). Nova Science Publishers Inc. https://hal.univ‐lorraine.fr/hal‐01780587

[psyp14706-bib-0034] Hämäläinen, H. , Kekoni, J. , Sams, M. , Reinikainen, K. , & Näätänen, R. (1990). Human somatosensory evoked potentials to mechanical pulses and vibration: Contributions of SI and SII somatosensory cortices to P50 and P100 components. Electroencephalography and Clinical Neurophysiology, 75(1), 13–21. 10.1016/0013-4694(90)90148-D 1688769

[psyp14706-bib-0035] Hansen, J. C. , & Hillyard, S. A. (1980). Endogenous brain potentials associated with selective auditory attention. Electroencephalography and Clinical Neurophysiology, 49(3–4), 277–290. 10.1016/0013-4694(80)90222-9 6158404

[psyp14706-bib-0036] Herrmann, C. S. , & Knight, R. T. (2001). Mechanisms of human attention: Event‐related potentials and oscillations. Neuroscience and Biobehavioral Reviews, 25(6), 465–476. 10.1016/S0149-7634(01)00027-6 11595268

[psyp14706-bib-0037] Hillyard, S. A. , & Anllo‐Vento, L. (1998). Event‐related brain potentials in the study of visual selective attention. Proceedings of the National Academy of Sciences, 95(3), 781–787. 10.1073/pnas.95.3.781 PMC33798 9448241

[psyp14706-bib-0038] Holstein, D. H. , Vollenweider, F. X. , Jäncke, L. , Schopper, C. , & Csomor, P. A. (2010). P50 suppression, prepulse inhibition, and startle reactivity in the same patient cohort suffering from posttraumatic stress disorder. Journal of Affective Disorders, 126(1–2), 188–197. 10.1016/J.JAD.2010.02.122 20347156

[psyp14706-bib-0039] Hunter, M. , Villarreal, G. , McHaffie, G. R. , Jimenez, B. , Smith, A. K. , Calais, L. A. , Hanlon, F. , Thoma, R. J. , & Cañive, J. M. (2011). Lateralized abnormalities in auditory M50 sensory gating and STG cortical thickness in PTSD: Preliminary results. Psychiatry Research: Neuroimaging, 191(2), 138–144. 10.1016/J.PSCYCHRESNS.2010.09.012 PMC4356025 21211947

[psyp14706-bib-0040] Kassambara, A. (2012). rstatix: Pipe‐Friendly Framework for Basic Statistical Tests. R Package version 0.7.0. https://cran.r‐project.org/web/packages/rstatix/index.html

[psyp14706-bib-0041] Kuznetsova, A. , Brockhoff, P. B. , & Christensen, R. H. B. (2017). lmerTest package: Tests in linear mixed effects models. Journal of Statistical Software, 82(13), 1–26. 10.18637/JSS.V082.I13

[psyp14706-bib-0042] Lenth, R. (2022). Emmeans: Estimated marginal means, aka least‐squares means. R Package Version 1.7.5. https://cran.r‐project.org/web/packages/emmeans/emmeans.pdf

[psyp14706-bib-0043] Lopez‐Calderon, J. , & Luck, S. J. (2014). ERPLAB: An open‐source toolbox for the analysis of event‐related potentials. Frontiers in Human Neuroscience, 8, 213. 10.3389/fnhum.2014.00213 24782741 PMC3995046

[psyp14706-bib-0044] Ludewig, S. , Ludewig, K. , Geyer, M. A. , Hell, D. , & Vollenweider, F. X. (2002). Prepulse inhibition deficits in patients with panic disorder. Depression and Anxiety, 15(2), 55–60. 10.1002/DA.10026 11891993

[psyp14706-bib-0045] Miyake, A. , Friedman, N. P. , Emerson, M. J. , Witzki, A. H. , Howerter, A. , & Wager, T. D. (2000). The unity and diversity of executive functions and their contributions to complex “frontal lobe” tasks: A latent variable analysis. Cognitive Psychology, 41(1), 49–100. 10.1006/COGP.1999.0734 10945922

[psyp14706-bib-0046] Näätänen, R. (1982). Processing negativity: An evoked‐potential reflection of selective attention. Psychological Bulletin, 92(3), 605–640. 10.1037/0033-2909.92.3.605 7156260

[psyp14706-bib-0047] Nieuwenhuis, S. , Aston‐Jones, G. , & Cohen, J. D. (2005). Decision making, the P3, and the locus coeruleus‐norepinephrine system. Psychological Bulletin, 131(4), 510–532. 10.1037/0033-2909.131.4.510 16060800

[psyp14706-bib-0048] Oldfield, R. C. (1971). The assessment and analysis of handedness: The Edinburgh inventory. Neuropsychologia, 9, 97–113. 10.1016/0028-3932(71)90067-4 5146491

[psyp14706-bib-0049] Pinto, N. F. C. , Duarte, M. , Gonçalves, H. , Silva, R. , Gama, J. , & Pato, M. V. (2021). Theta‐burst stimulation is able to impact cognitive processing: A P300 and neuropsychological test study. Neuropsychobiology, 80(4), 288–298. 10.1159/000511605 33395687

[psyp14706-bib-0050] Polich, J. (2007). Updating P300: An integrative theory of P3a and P3b. Clinical Neurophysiology, 118(10), 2128–2148. 10.1016/j.clinph.2007.04.019 17573239 PMC2715154

[psyp14706-bib-0051] Qi, S. , Ding, C. , & Li, H. (2013). Neural correlates of inefficient filtering of emotionally neutral distractors from working memory in trait anxiety. Cognitive, Affective, & Behavioral Neuroscience, 14, 253–265. 10.3758/s13415-013-0203-5 23963822

[psyp14706-bib-0052] R Core Team . (2021). R: A language and environment for statistical computing. R Foundation for Statistical Computing, Vienna, Austria. https://www.R‐project.org/

[psyp14706-bib-0053] Ross, J. A. , & Van Bockstaele, E. J. (2021). The locus Coeruleus‐ norepinephrine system in stress and arousal: Unraveling historical, current, and future perspectives. Frontiers in Psychiatry, 11, 601519. 10.3389/fpsyt.2020.601519 33584368 PMC7873441

[psyp14706-bib-0054] Rossi, S. , Bartalini, S. , Ulivelli, M. , Mantovani, A. , Di Muro, A. , Goracci, A. , Castrogiovanni, P. , Battistini, N. , & Passero, S. (2005). Hypofunctioning of sensory gating mechanisms in patients with obsessive‐compulsive disorder. Biological Psychiatry, 57(1), 16–20. 10.1016/j.biopsych.2004.09.023 15607295

[psyp14706-bib-0055] RStudio Team . (2022). RStudio: Integrated development environment for R (2022.7.1.554). RStudio.

[psyp14706-bib-0056] Sehlmeyer, C. , Konrad, C. , Zwitserlood, P. , Arolt, V. , Falkenstein, M. , & Beste, C. (2010). ERP indices for response inhibition are related to anxiety‐related personality traits. Neuropsychologia, 48(9), 2488–2495. 10.1016/j.neuropsychologia.2010.04.022 20434466

[psyp14706-bib-0057] Spielberger, C. D. , Gorsuch, R. L. , Lushene, R. , Vagg, P. R. , & Jacobs, G. A. (1983). Manual for the state‐trait anxiety inventory. Consulting Psychologists.

[psyp14706-bib-0058] Stout, D. M. , Shackman, A. J. , & Larson, C. L. (2013). Failure to filter: Anxious individuals show inefficient gating of threat from working memory. Frontiers in Human Neuroscience, 7(58), 1–10. 10.3389/fnhum.2013.00058 23459454 PMC3586709

[psyp14706-bib-0059] Vandoolaeghe, E. , van Hunsel, F. , Nuyten, D. , & Maes, M. (1998). Auditory event related potentials in major depression: Prolonged P300 latency and increased P200 amplitude. Journal of Affective Disorders, 48(2–3), 105–113. 10.1016/s0165-0327(97)00165-1 9543199

[psyp14706-bib-0060] Veale, J. F. (2014). Edinburgh handedness inventory ‐ short form: A revised version based on confirmatory factor analysis. Laterality, 19, 164–177. 10.1080/1357650X.2013.783045 23659650

[psyp14706-bib-0061] Xia, L. , Mo, L. , Wang, J. , Zhang, W. , & Zhang, D. (2020). Trait anxiety attenuates response inhibition: Evidence from an ERP study using the go/NoGo task. Frontiers in Behavioral Neuroscience, 14(28), 1–9. 10.3389/fnbeh.2020.00028 32218724 PMC7078641

[psyp14706-bib-0062] Xiu, B. , Andanty, C. , Dai, N. , Zai, C. C. , Graff, A. , McNeely, H. , Daskalakis, Z. J. , & De Luca, V. (2022). Association between the visual N1‐P2 complex and neuroticism. Clinical EEG and Neuroscience, 53(2), 95–103. 10.1177/15500594211039937 34515573

[psyp14706-bib-0063] Yamaguchi, S. , & Knight, R. T. (1990). Gating of somatosensory input by human prefrontal cortex. Brain Research, 521(1–2), 281–288. 10.1016/0006-8993(90)91553-S 2207666

